# Fast Reaction Kinetics via Interfacial Mediation in Quasi- and All-Solid-State Lithium-Sulfur Batteries

**DOI:** 10.34133/research.0949

**Published:** 2025-10-10

**Authors:** Ke Wang, Yanjiao Ma, Torsten Brezesinski, Yuan Ma, Yuping Wu

**Affiliations:** ^1^ Confucius Energy Storage Lab, School of Energy and Environment & Z Energy Storage Center, Southeast University, Nanjing 211189, China.; ^2^ School of Energy and Mechanical Engineering, Nanjing Normal University, Nanjing 210023, China.; ^3^ Institute of Nanotechnology, Karlsruhe Institute of Technology, 76131 Karlsruhe, Germany.

## Abstract

In recent years, lithium-sulfur batteries have attracted much interest owing to the natural abundance of sulfur and its high theoretical specific capacity (*q*_th_ ≈ 1,672 mAh g^−1^), offering the potential to achieve cell-level energy densities exceeding 400 Wh kg^−1^. While excess electrolyte facilitates redox reactions, it compromises specific energy and safety, driving the shift toward lean-electrolyte and solid-state systems. Although this helps suppress polysulfide shuttling, such strategies suffer from sluggish solid–solid conversion reactions and poor interfacial kinetics. Recently, studies adopting interfacial mediator strategies have emerged to address these challenges by enabling localized redox reactions at otherwise inactive interfaces. This perspective highlights advances in mediator-facilitated sulfur conversion under quasi- and all-solid-state conditions, offering insights into designing high-performance (electrolyte-efficient) lithium-sulfur batteries.

Lithium-sulfur (Li-S) batteries utilizing cost-effective, high-capacity sulfur cathodes are regarded as promising candidates for next-generation electrochemical energy storage [1–3]. However, the conversion relies on soluble lithium polysulfides (LiPSs), whose dissolution, shuttling, and side reactions with lithium cause active material loss and capacity fading. Mitigation includes immobilizing LiPSs in the cathode (with a weight penalty) or suppressing their solubility by tailoring electrolyte solvation [4–6]. A more radical route is to adopt a solid–solid-type conversion that bypasses the solution pathway and decouples the reactions from the bulk electrolyte. Nevertheless, these strategies often suffer from compromised kinetics and limited practical applicability [7–9].

Recently, Liu et al. [10] and Song et al. [11] enhanced reaction rates significantly by introducing active mediators at the cathode interface to promote localized surface reactions (see Fig. 1 for a schematic illustration of the general concept). These studies exemplify how mediator strategies can accelerate sulfur conversion, providing a set of general design principles for effective mediators in Li-S batteries. Two prerequisites must be satisfied: (a) the redox potential properly matching the targeted active species to enable the desired charge transfer and (b) recyclability to ensure feasibility and continuity. Beyond these common requirements, the design principles diverge depending on electrolyte content. In liquid-rich systems, the priority is to suppress LiPS shuttling. With reduced electrolyte, the shuttle is alleviated, and the focus shifts to accelerating reaction kinetics. In the absence of liquid electrolyte, LiPSs are no longer involved, and mediators are instead designed to facilitate fast charge transfer across solid interfaces [12–14].

**Fig. 1.**
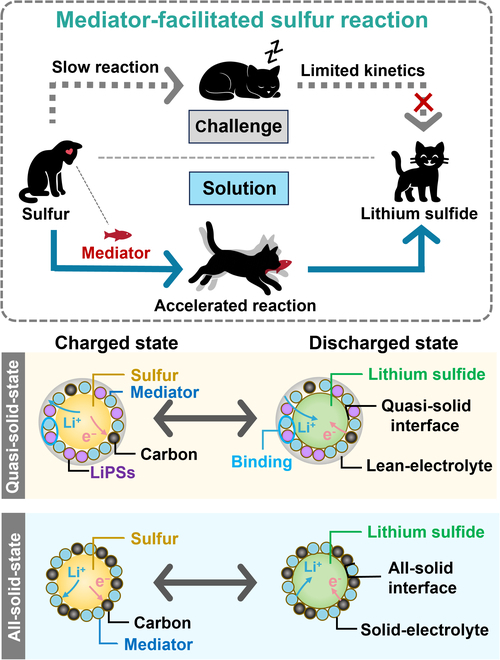
Schematic illustration of mediator-enabled activation in sulfur conversion reactions. LiPSs, lithium polysulfides.

This kind of mediation concept has been demonstrated in quasi-solid-state Li-S batteries using lean-electrolyte systems. Specifically, the redox-active, organic mediator introduced into the low-viscosity, ether-based electrolyte can reversibly coordinate with (quasi-solid) Li_2_S*_x_* species, thereby dynamically generating transient, surface-localized solvation domains. These domains enable the conversion to proceed via faster solution-phase pathways while suppressing bulk polysulfide dissolution [10]. Mechanistically, the mediator interacts directly with both soluble and insoluble sulfur species, forming reversible coordination complexes that chemically bridge otherwise isolated Li_2_S/Li_2_S*_x_* domains. Through this process, the spatial continuity of redox-active regions is enhanced, ultimately lowering the local kinetic barrier for electron/ion access. At the same time, the mediator operates within a low-permittivity, shuttle-suppressing solvent matrix, which helps confine activity to the electrode interface and prevents long-range polysulfide migration. This design exemplifies how controlled interfacial solvation and molecular-level redox mediation can be co-engineered to unlock fast reaction pathways in systems that are commonly constrained by sluggish transport and electrochemical passivation. When assembled into pouch cells, the system achieved about 330 Wh kg^−1^ at a 2.4-Ah total capacity, demonstrating not only promising specific energy but also practical scalability.

In quasi-solid-state systems, mediators regulate the LiPSs within localized dissolution environments. Building on this, the all-solid-state concept is bypassing the solution-mediated pathway entirely, with mediators tailored to accelerate sulfur conversion. To overcome the intrinsic challenges, a glassy lithium thioborophosphate iodide (LBPSI) solid electrolyte (SE) has been developed, aiming to promote redox reactions at otherwise inactive SE|Li_2_S 2-phase boundaries [11]. Unlike conventional ion conductors, LBPSI incorporates redox-active iodine species, which undergo reversible electrochemical cycling (I^−^ ⇌ I_2_/I_3_^−^) and serve as dynamic surface mediators that chemically activate solid–solid conversion reactions. During charging, iodine anions present on the SE surface are electrochemically oxidized to I_2_/I_3_^−^ at the SE|C interface; these oxidized species then diffuse locally and chemically oxidize adjacent Li_2_S particles in direct contact with the SE. In this manner, the mediator bypasses the requirement for rare SE|Li_2_S|C triple-phase boundaries and instead enables reactions to proceed at the far more abundant SE|Li_2_S 2-phase boundaries, which are otherwise electrochemically inert. The glass-forming tendency of LBPSI suppresses crystallization and allows the reversible I^−^/I_2_/I_3_^−^ cycling to occur without irreversible electrolyte degradation. This mechanism enables excellent electrochemical performance across a broad range of operating conditions. Specifically, the all-solid-state Li-S batteries delivered a high specific capacity of 1,497 mAh g^−1^_sulfur_ at 2 C (with 1 C = 1,672 mA g^−1^) and 30 °C, demonstrating good rate capability. Furthermore, they exhibited a notable longevity, retaining about 80% of their initial capacity after more than 25,000 cycles at 5 C and 25 °C.

Of note, similar interfacial mediation strategies have been independently reported in other studies, emphasizing the broader applicability of the approach. For instance, in Li–CO_2_ batteries, Li et al.’s team [15] used a solid-state Cu(II)/Cu(I) redox mediator to activate CO_2_ and promote Li_2_C_2_O_2_ formation, realizing efficient and stable cycling. Such mediator strategies have also inspired advances in other electrochemical and catalytic contexts. Examples include C–C coupling electrocatalysis and the design of hydrogel-based nanozymes, illustrating their potential well beyond energy storage [16,17]. These studies underscore the growing interest in using redox mediators, pointing to a universal strategy that enables interfacial redox activity beyond the conventional limitations of the field.
